# Suppressive Effect and Molecular Mechanism of *Houttuynia cordata* Thunb. Extract against Prostate Carcinogenesis and Castration-Resistant Prostate Cancer

**DOI:** 10.3390/cancers13143403

**Published:** 2021-07-07

**Authors:** Subhawat Subhawa, Aya Naiki-Ito, Hiroyuki Kato, Taku Naiki, Masayuki Komura, Aya Nagano-Matsuo, Ranchana Yeewa, Shingo Inaguma, Teera Chewonarin, Ratana Banjerdpongchai, Satoru Takahashi

**Affiliations:** 1 Department of Experimental Pathology and Tumor Biology, Nagoya City University Graduate School of Medical Sciences, 1-Kawasumi, Mizuho-cho, Mizuho-ku, Nagoya 467-8601, Japan; subhawat_s@cmu.ac.th (S.S.); h.kato@med.nagoya-cu.ac.jp (H.K.); naiki@med.nagoya-cu.ac.jp (T.N.); komura@med.nagoya-cu.ac.jp (M.K.); aya.ngn@med.nagoya-cu.ac.jp (A.N.-M.); yeewa.ranchana@gmail.com (R.Y.); inaguma@med.nagoya-cu.ac.jp (S.I.); sattak@med.nagoya-cu.ac.jp (S.T.); 2 Department of Biochemistry, Faculty of Medicine, Chiang Mai University, 110 Intravaroros Rd., Sripoom, Muang, Chiang Mai 50200, Thailand; teera.c@cmu.ac.th

**Keywords:** *Houttuynia cordata* Thunb., natural extracts, functional food, anti-proliferation, apoptosis, prostate cancer, anti-migration

## Abstract

**Simple Summary:**

This study explored the chemopreventive effects of *Houttuynia cordata* Thunb. (HCT) extracts against prostate carcinogenesis in both androgen-sensitive prostate cancer and castration-resistant prostate cancer (CRPC) using the Transgenic Rat for Adenocarcinoma of Prostate (TRAP) model, CRPC xenograft mice, and prostate cancer cell lines. HCT suppressed cell proliferation and stimulated apoptosis via inactivation of AKT/ERK/MAPK in both androgen-sensitive prostate cancer and CRPC cell lines. HCT also inhibited cell migration and EMT phenotypes through the STAT3/Snail/Twist pathway. One of the active compounds of HCT was identified as rutin. Consistent with in vitro study, the incidence of adenocarcinoma in the TRAP model and CRPC tumor growth in the xenograft model were suppressed by induction of apoptosis and inactivation of AKT/ERK/MAPK by HCT intake. Our data demonstrated that HCT attenuated androgen-sensitive prostate cancer and CRPC by mechanisms that may involve inhibition of cell growth and caspase-dependent apoptosis pathways.

**Abstract:**

*Houttuynia cordata* Thunb. (HCT) is a well-known Asian medicinal plant with biological activities used in the treatment of many diseases including cancer. This study investigated the effects of HCT extract and its ethyl acetate fraction (EA) on prostate carcinogenesis and castration-resistant prostate cancer (CRPC). HCT and EA induced apoptosis in androgen-sensitive prostate cancer cells (LNCaP) and CRPC cells (PCai1) through activation of caspases, down-regulation of androgen receptor, and inactivation of AKT/ERK/MAPK signaling. Rutin was found to be a major component in HCT (44.00 ± 5.61 mg/g) and EA (81.34 ± 5.21 mg/g) in a previous study. Rutin had similar effects to HCT/EA on LNCaP cells and was considered to be one of the active compounds. Moreover, HCT/EA inhibited cell migration and epithelial-mesenchymal transition phenotypes via STAT3/Snail/Twist pathways in LNCaP cells. The consumption of 1% HCT-mixed diet significantly decreased the incidence of adenocarcinoma in the lateral prostate lobe of the Transgenic rat for adenocarcinoma of prostate model. Similarly, tumor growth of PCai1 xenografts was significantly suppressed by 1% HCT treatment. HCT also induced caspase-dependent apoptosis via AKT inactivation in both in vivo models. Together, the results of in vitro and in vivo studies indicate that HCT has inhibitory effects against prostate carcinogenesis and CRPC. This plant therefore should receive more attention as a source for the future development of non-toxic chemopreventive agents against various cancers.

## 1. Introduction

Prostate cancer is the second most frequent malignancy in men worldwide [1]. Its incidence and mortality rates are strongly correlated with rising age, with the average age range significantly greater than 65 years [2]. Prostate cancer progression and growth depend on androgens and can be inhibited by androgen deprivation therapy (ADT) [3,4,5]. However, prostate cancer relapses in an androgen-independent form and progresses into castration-resistant prostate cancer (CRPC) within two years with highly metastatic advanced diseases [6]. In addition, the activation of a ligand-independent androgen receptor (AR) is known as an outlaw pathway, or non-genomic AR signaling, which stimulates AR phosphorylation by either the AKT (protein kinase B) or the mitogen-activated protein kinase (MAPK) pathways [7,8,9,10]. Inhibition of these pathways is the objective for prevention of the prostate carcinogenesis and CRPC.

Herbs contain many nontoxic natural compounds and various combinations have been used in studies on the prevention of multistage carcinogenesis [11]. Herbs have been widely used in the formulation of dietary supplements for health maintenance, and the number of such products in the marketplace has been steadily rising. With regards to their phytochemical compositions, herbs consist of various phytochemicals in combination, and they are more used as a crude extract than as each isolated constituent. Therefore, the herb should be tested and approved based on scientific evidence of efficiency for cancer prevention in plant-based foods [12]. Consuming a diet rich in phenolic acids, such as fruits and vegetables, is related to a reduced risk of prostate cancer progression. For example, phenolic acids and flavonoids induce apoptosis by increased expression of pro-apoptotic proteins and suppressed expression of anti-apoptotic proteins in in vitro study [13,14]. Some dietary compounds show anti-cancer properties against prostate cancers through inhibition of proliferation and metastasis [15].

*Houttuynia cordata* Thunb. (HCT) is a common herbal medicine plant found in East Asia. There are many phytochemicals in HCT such as phenolic acids, flavonoids, and alkaloids [16]. In our recent study, HCT showed effective cytotoxicity against breast cancer cells by inducing caspase-dependent apoptosis [17] and it is probable it inhibits benign prostatic hyperplasia (BPH) by blocking the binding of androgen to its receptor in mice [18]. Therefore, it is possible that HCT could be used as a functional natural product to prevent carcinogenesis. However, the chemopreventive effect of HCT on prostate cancer has not yet been established.

We previously developed a Transgenic Rat for Adenocarcinoma of Prostate (TRAP) model, which involves induction of prostate adenocarcinoma under androgen-sensitive conditions over a short time, and which is useful for analyzing chemopreventive effects on prostate carcinogenesis [19,20,21,22,23]. Furthermore, the androgen-independent prostate cancer cell line, PCai1, which was derived from TRAP tumor, and the subcutaneous xenograft of PCai1 cells in mice can be used as model of CRPC [24,25]. Consequently, these models are suitable for clarifying the effect of HCT on prostate cancer [26,27].

This study aimed to investigate the chemopreventive effect of HCT extract on prostate carcinogenesis using the TRAP model, and examined the anti-proliferation and anti-migration effects, as well as the underlying molecular mechanisms, in LNCaP cells. Furthermore, the inhibitory effect of HCT on CRPC was elucidated using a PCai1 xenograft mouse model and the underlying mechanisms were explored in PCai1 cells.

## 2. Materials and Methods

### 2.1. Cell Culture

Phosphate-buffered saline (PBS), rutin (purity ≥ 90%), and trypsin-EDTA solution were purchased from Fujifilm Wako Pure Chemical Corporation (Osaka, Japan). Dimethyl sulfoxide (DMSO) and chlorogenic acid (purity ≥ 95%) were purchased from Sigma–Aldrich Chemical, Inc. (St Louis, MO, USA). The human prostate cancer cell lines, LNCaP, were purchased from the American Type Culture Collection (Manassas, Virginia). Both LNCaP and the original rat CRPC cell line, PCai1, were cultured in RPMI-1640 medium and supplemented with 10% fetal bovine serum (FBS) Dominican Republic (Biosera, Manila, Philippines), at 37 °C under a 5% CO_2_ atmosphere. The cells were harvested and then plated, or sub-cultured when they obtained 70% to 80% confluence for preservation or cycle passages.

### 2.2. Plant Sample Preparation

HCT collected from Mueang, Lamphun, Thailand (Prolac Corporation, Ltd.) was roughly blended and then extracted in 80% ethanol (ratio 1:10 *w*/*v*) by stirring overnight, as described in a previous study [17,28]. The supernatant from the extraction was directly filtered through Whatman filter paper No. 1; the fraction was partially partitioned with hexane (ratio 1:1) and evaporated using a rotating evaporator (40 °C) at 100–150 mbar. After that, the fraction was lyophilized and formed as a powder part of the solute in hexane (H). The ethyl acetate fraction of HCT (EA) was prepared by further mixing a part of the hexane insoluble fraction with ethyl acetate (ratio 1:1) and was separated and then evaporated under vacuum until all of the solvents were removed and lyophilized to produce the EA powder part. Another aqueous fraction was evaporated and lyophilized under the same conditions to produce a powder part of residue fraction (R).

### 2.3. Identification of Phytochemicals by HPLC

All extracts, including HCT, EA, H, and R, were analyzed using high-performance liquid chromatography (HPLC), as in a previous study [17]. Briefly, a Phenomenex RP-Gemini NX C18 (250 mm × 4.6 mm, 5 μm) HPLC column was used. Mobile phases were 0.1% trifluoroacetic acid (TFA): Water as solvent A and methanol as solvent B at a flow rate of 1 mL/min were controlled using a gradient elution program. Ten μL of each sample was injected into the column with a flow rate of 1.0 mL/min and monitored at 280 and 293 nm, respectively.

### 2.4. Cell Viability Assay

Cell viability was tested by trypan blue staining [29]. Briefly, 2 × 10_4_ cells/well were seeded in a 24-well plate and incubated overnight at 37 °C under 5% CO_2_. Subsequently, HCT, EA, rutin, or chlorogenic acid (0–200 μg/mL) were added to LNCaP and PCai1 cells. After 48 h, the trypsinized cells were suspended in media and stained with equal parts of 0.4% trypan blue dye. Cell viability in each well was determined and the values were compared with the control vehicle (0 μg/mL) and averaged from three independent experiments.

### 2.5. Cell Cycle Assay

Cell cycle arrest was investigated using Guava^®^ easyCyte flow cytometers with Guava InCyte^TM^ software (EDM Millipore Corp., Billerica, MA, USA) [26]. Briefly, cancer cells were seeded in six-well plates and then cultured with the herbal extracts (0–200 μg/mL) in conditioned media for 48 h. The cells were then collected and fixed in 70% ethanol overnight and stained with Guava^®^ Cell Cycle reagent (EDM Millipore Corp.). In each experiment, determinations were conducted in triplicate from three independent experiments.

### 2.6. Apoptosis Assay

LNCaP and PCai1 cells were incubated with HCT, EA, rutin, or chlorogenic acid for 48 h. According to the Guava Nexin Assay protocol, the cells were stained with annexin V-PE and 7-amino actinomycin D (7-AAD) (Guava^®^ Nexin Reagent, Luminex Corporation, Austin, TX, USA) for 20 min. Apoptosis was evaluated on a Guava PCA Instrument using Guava^®^ Viacount^TM^ Software (EDM Millipore Corp.) [17]. In each experiment, determinations were conducted in triplicate from three independent experiments.

### 2.7. Western Blotting

The treated cells and frozen tissues were lysed with a RIPA buffer containing a protease inhibitor cocktail tablet (Roche, Mannheim, Germany) and the amount of protein was measured using a Bradford assay kit, as described in a previous study [17]. To determine protein expression, protein samples were run on SDS-PAGE and then transferred onto the nitrocellulose membranes. After that, 5% skim milk diluted in 0.1% Tween-TBS was used to block non-specific binding on the membrane for 1 h at room temperature. The membrane was incubated with the individual specific primary antibodies, at 4 °C, overnight with cyclin D1, p21, cleaved caspase-3, caspase-3, cleaved caspase-7, caspase-7, protein kinase B (AKT), phospho-AKT, p38 MAPK, phospho-p38 MAPK, extracellular signal-regulated kinase (ERK)1/2, phospho-ERK1/2, poly (ADP-ribose) polymerase (PARP), N-cadherin, STAT3, phosphor-STAT3, and Snail, which were purchased from Cell Signaling Technology (Danvers, MA, USA); E-cadherin and CDK4 (Thermo Fisher Scientific, Waltham, MA, USA); vimentin and Twist (Abcam, Cambridge, UK); AR and β-actin were obtained from Sigma–Aldrich Chemical, Inc. The membrane was washed and incubated with peroxidase-labeled secondary antibodies, including anti-rabbit IgG and anti-mouse IgG (Thermo Fisher Scientific), for 2 h at room temperature and then applied with ImmunoStar^®^ Zeta (Fujifilm Wako Pure Chemical Corporation). The intensity of each band was measured using ImageJ software (National Institute of Health, Bethesda, MD, USA).

### 2.8. Wound-Healing Assay

Briefly, LNCaP cells (1 × 10^6^ per well) were plated in six-well plates, cultured to 100% confluence, scraped off using a pipette tip, and washed out gently with serum-free medium. Both HCT (0–100 μg/mL) and EA (0–50 μg/mL) were added and incubated for 24 h, as described previously [17]. The percentage of the cell-deficient area was determined at 0, 12, and 24 h under a Nikon Eclipse Ts2 phase-contrast microscope (Nikon Corporation, Tokyo, Japan). ImageJ was used to determine the migration distance.

### 2.9. TRAP Model Experimental Protocol

Six-week-old male TRAP rats were randomly divided into three groups and received either control diet (n = 13) or 0.2% (n = 12) or 1% (n = 12) HCT mixed diet for 10 weeks. At the end of the experiment, rats were sacrificed and blood, livers, kidneys, and prostate glands were collected. Blood was centrifuged and serum was collected for determination of testosterone and 17 β-estradiol serum levels using an enzyme-linked immunosorbent assay kit (Abcam) according to the manufacturer’s protocol. Livers and kidneys were fixed in 10% formalin, while lateral and ventral lobes of each rat prostate were divided into two parts: The first part was frozen in liquid nitrogen and stored at −80 °C for Western blotting, and the other part was fixed in 10% formalin and used for immunohistochemistry. Another portion of prostate part was fixed in 10% formalin for paraffin-embedded sections. The paraffin blocks were sectioned and stained with hematoxylin and eosin (H&E). The incidence of adenocarcinoma and the relative percentage of neoplastic lesions were quantified using H&E-stained slides, as previously described [20,30]. Briefly, neoplastic lesions were classified as low-grade prostatic intraepithelial neoplasia (LG-PIN), HG-PIN, or adenocarcinoma. The percentage of neoplastic lesion in each type of acini was calculated based on the total number of acini in each prostatic lobe. Experiments were approved by the Institutional Animal Care and Use Committee at Nagoya City University School of Medical Sciences, Nagoya, Japan (no. 20-001, approved on 2 June 2020).

### 2.10. Xenograft Study

The effect of crude HCT extract on prostate tumorigenesis was investigated in a PCai1 xenograft model. PCai1 cells (1 × 10^6^) were injected subcutaneously in seven-week-old male KSN/nunu mice. After one week, the mice were randomly separated into three groups (N = 15) to receive (i) basal diet, (ii) 0.2% HCT mixed diet, or (iii) 1% HCT mixed diet. Food consumption was measured every three days, while body weight and tumor size were measured once a week. Tumor volume was calculated as follows: 0.52 × length × width × height (in millimeters). Mice were sacrificed four weeks after treatment. Some organs such as liver and kidneys were collected, weighed, and fixed in 10% buffered formalin. All tumors were collected and divided into two parts: The first part was frozen in liquid nitrogen and stored at −80 °C until processed, and the other part was fixed with 10% buffered formalin and used for immunohistochemistry. Experiments were approved by the Institutional Animal Care and Use Committee at Nagoya City University School of Medical Sciences, Nagoya, Japan (no. 19-043, approved on 8 April 2020).

### 2.11. Immunohistochemistry

Organs were collected, weighed, fixed in 10% formalin and prepared for paraffin embedding. The paraffin blocks were sectioned and incubated with antibodies against AR (Sigma–Aldrich Chemical, Inc.), Ki-67 (SP6; Acris Antibodies GmbH, Herford, Germany), SV40T antigen (PharMingen, CA, USA), or CD31 (Abcam) as previously described [31]. To detect apoptotic cells, a TUNEL assay using an in situ apoptosis detection kit was performed according to the manufacturer’s protocol (Takara, Otsu, Japan).

### 2.12. Statistical Analysis

The data were expressed as the mean ± standard deviation (SD). All statistical analyses were analyzed by using one-way ANOVA with: LSD or Tukey’s post hoc test depending on the experiments. All data was analyzed using GraphPad Prism 8.0 software (GraphPad Software, Inc., San Diego, CA, USA). *p* < 0.05 was considered statistically significant.

## 3. Results

### 3.1. Comparison of Phytochemical Compositions in HCT and EA by HPLC

**Figure 1:**
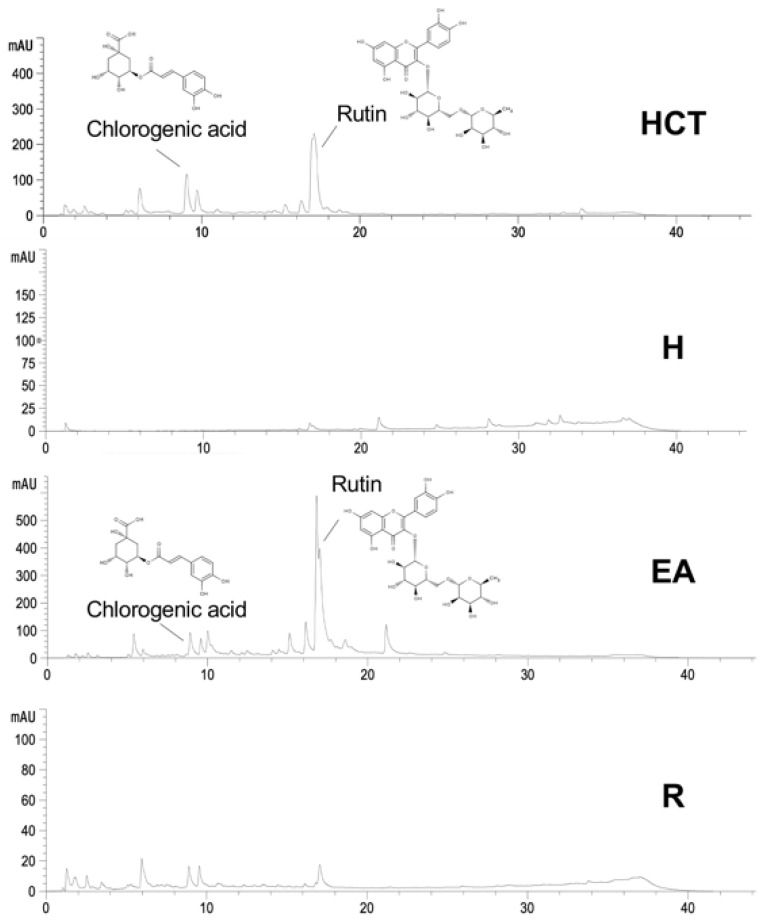
The level of rutin and chlorogenic acid of ethanolic *H. cordata* extract compared to hexane (H), ethyl acetate (EA), and residue fractions (R).

We investigated the HPLC chromatograms of HCT crude extract compared to all fractions, including H, EA, and R fractions. In our recent study, the major flavonoid and phenolic acid compounds in HCT extract were rutin and chlorogenic acid, respectively [17]. We then quantitatively compared the level of rutin, chlorogenic acid, and other components in all fractions of HCT (Figure 1). As shown in Table 1, HPLC analysis indicated that contents of rutin and chlorogenic acid in EA were also high among other flavonoid and phenolic acid compounds. The levels of chlorogenic acid and rutin in HCT were 25.50 ± 3.41 and 44.00 ± 5.61 mg/g extract, respectively, while their levels in EA were 15.15 ± 1.37 and 81.34 ± 5.21 mg/g extract, respectively (Table 1). The data assumes that rutin is enriched roughly two-fold in EA compared with HCT, while chlorogenic acid was slightly decreased in EA compared with HCT.

### 3.2. HCT and EA Inhibit Cell Proliferation and Induce Apoptosis in Prostate Cancer Cell Lines

**Figure 2:**
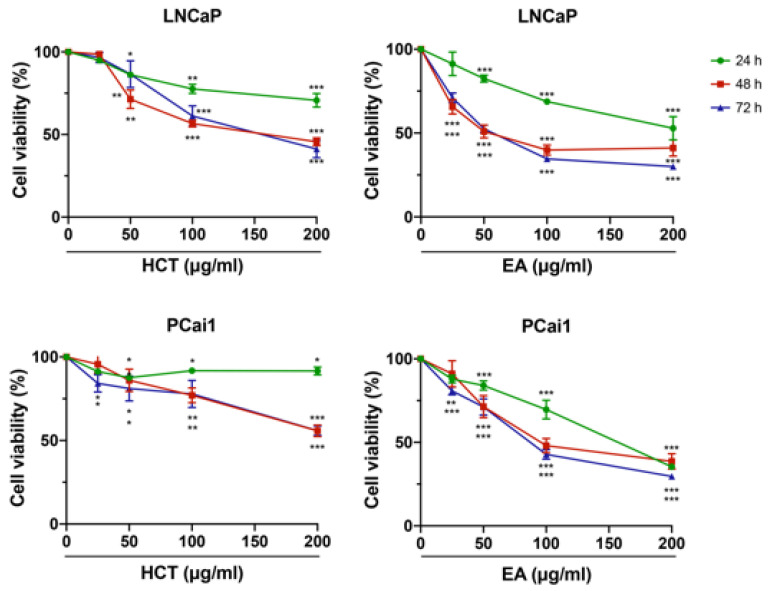
Cytotoxic effects of HCT and EA in prostate cancer cell lines. LNCaP and PCai1 cells were treated with HCT or EA (25–200 µg/mL) at 24, 48, and 72 h. The data represent three independent experiments. * *p* < 0.05; ** *p* < 0.01; *** *p* < 0.001 vs. control (one-way ANOVA with Tukey’s post hoc test).

Subsequent to our HPLC results, a further study was conducted to determine the cytotoxicity of EA in prostate cancer cells, as well as that of the crude extract, HCT. Prostate cancer cell lines, the androgen-sensitive human prostate cancer cell line, LNCaP, and the castration-resistant rat prostate cancer cell line, PCai1, were used. Trypan blue staining indicated that both HCT and EA decreased the cell viability of both LNCaP and PCai1 cells in a dose-dependent manner (Figure 2). The inhibitory effect of cell viability by HCT and EA at 24 h was generally weaker than that at 48 h and 72 h, while there was no difference in cell viability between 48 h and 72 h in both cells (Figure 2).

**Figure 3:**
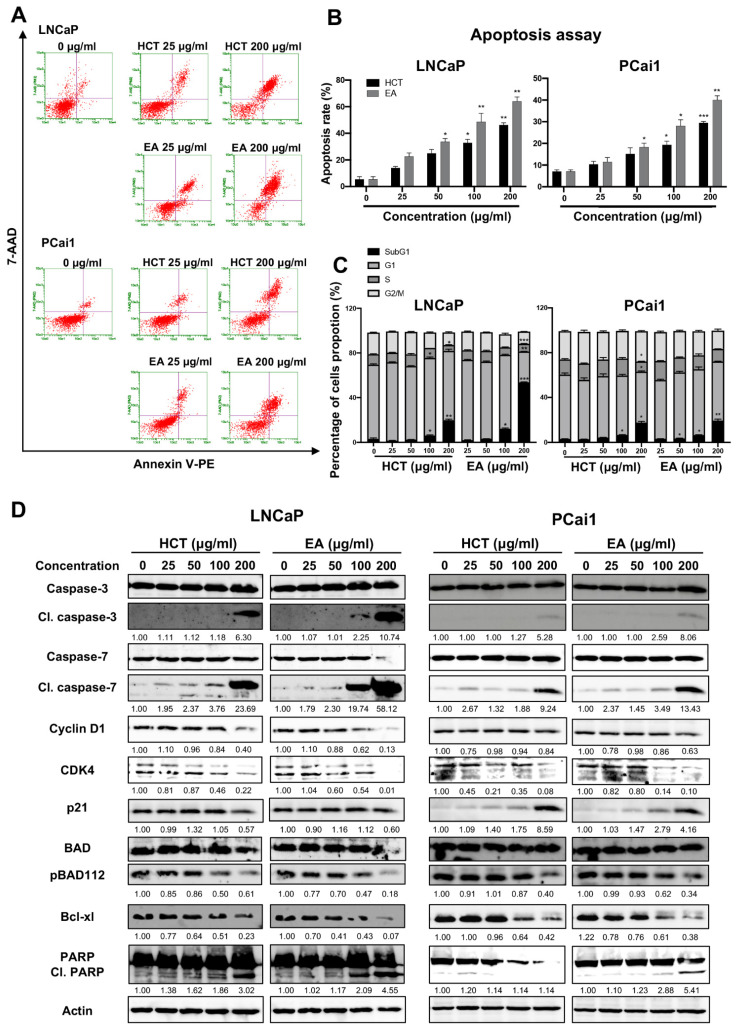
Apoptosis induction by HCT and EA in prostate cancer cell lines. Flow cytometric analysis of apoptosis induction in LNCaP and PCai1 cells treated with HCT and EA for 48 h (**A**). The percentages of apoptotic cells were quantitated using Guava^®^ ViacountTM Software (**B**). Effects of HCT and EA on the cell cycle of LNCaP and PCai1 cells for 48 h (**C**). Immunoblotting analysis to show the cell proliferation and apoptosis-related proteins in LNCaP and PCai1 cells in response to HCT and EA (**D**). Bar graphs showed the summarized data of three independent experiments and were performed in duplicate compared with the DMSO-treated control. The data represented the means ± SD. Whole un-cropped images see Figure S3. * *p* < 0.05, ** *p* < 0.01, and *** *p* < 0.001 vs. control. HCT, *Houttuynia cordata* Thunb.; EA, ethyl acetate fraction of *H. cordata*.

Additionally, the cytotoxic effect of EA was stronger than that of HCT in prostate cancer cells. Hence to further clarify these results, we then assessed the induction of apoptosis and performed cell cycle analysis using flow cytometry. As shown in Figure 3A–C, both HCT and EA significantly increased the percentage of apoptotic cells in both prostate cancer cell lines. Cell cycle assay indicated that HCT and EA significantly increased the population of cells in the sub-G1 phase, but there was no significant trend for other phases. The results were further confirmed by alteration of cell-cycle relevant and apoptosis-related protein expression. Interestingly, the decreased expression of cyclin D1 and CDK4 protein was observed, while p21 was increased by HCT and EA. Moreover, the expression of cleaved caspases-3 and -7, and cleaved PARP were noticeably elevated by both treatments. On the other hand, HCT and EA reduced the expression of anti-apoptotic proteins such as Bcl-xl and BH3-only Bcl-2 family protein, e.g., p-Bad (Figure 3D). These results suggest that both HCT and EA induce apoptosis in both LNCaP and PCai1 cell lines.

### 3.3. HCT and EA Induce Apoptosis via Suppressing AR Expression and Inactivating the Phosphorylation of AKT/ERK/p38 MAPK Pathways in Prostate Cancer Cells

**Figure 4:**
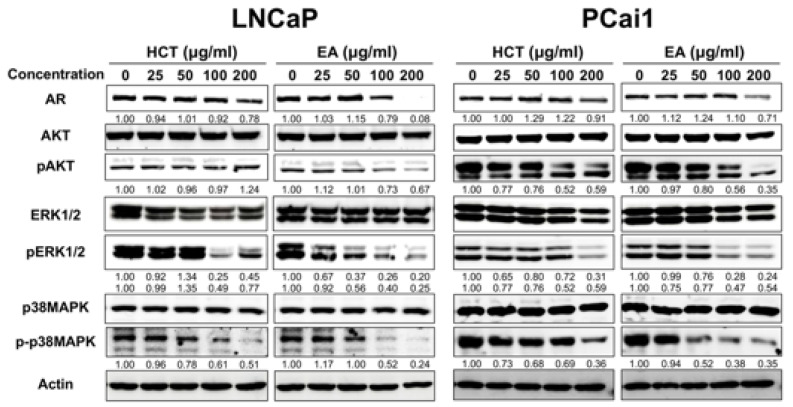
Western blot analysis of AKT, ERK, and MAPK pathways in HCT and EA-induced apoptosis of prostate cancer cells. The expression level of each protein expression in LNCaP and PCai1 cells treated with HCT and EA for 48 h and measured by western blotting. Whole un-cropped images see Figure S4. HCT, *Houttuynia cordata* Thunb.; EA, ethyl acetate fraction of *H. cordata*.

Dysregulation of apoptosis through abnormal AR signaling is involved in prostate cancer development in LNCaP [32]. Besides, AKT, ERK, and p38 MAPK, the possible candidates to control cell proliferation and apoptosis, are related to prostate cancer cell lines [33]. Therefore, we investigated the roles of these molecules on the induction of apoptosis by HCT and EA in LNCaP and PCai1 cells. Western blotting revealed that both HCT and EA significantly decreased the expression of AR, p-p38 MAPK, and p-ERK1/2 in both prostate cancer cell lines (Figure 4). Inactivation of AKT by EA was observed in both LNCaP and PCai1 cells, while HCT decreased p-AKT only in PCai1 cells. These results indicate that the effect of HCT and EA in LNCaP and PCai1 are related to the decreased expression of the AR and inactivation of AKT, ERK, and p38 MAPK signaling pathways, leading to the stimulation of apoptosis in androgen-sensitive prostate cancer and CRPC cells.

### 3.4. Rutin Is a Responsible Component of HCT to Induce Apoptosis in LNCaP Cell Lines

**Figure 5:**
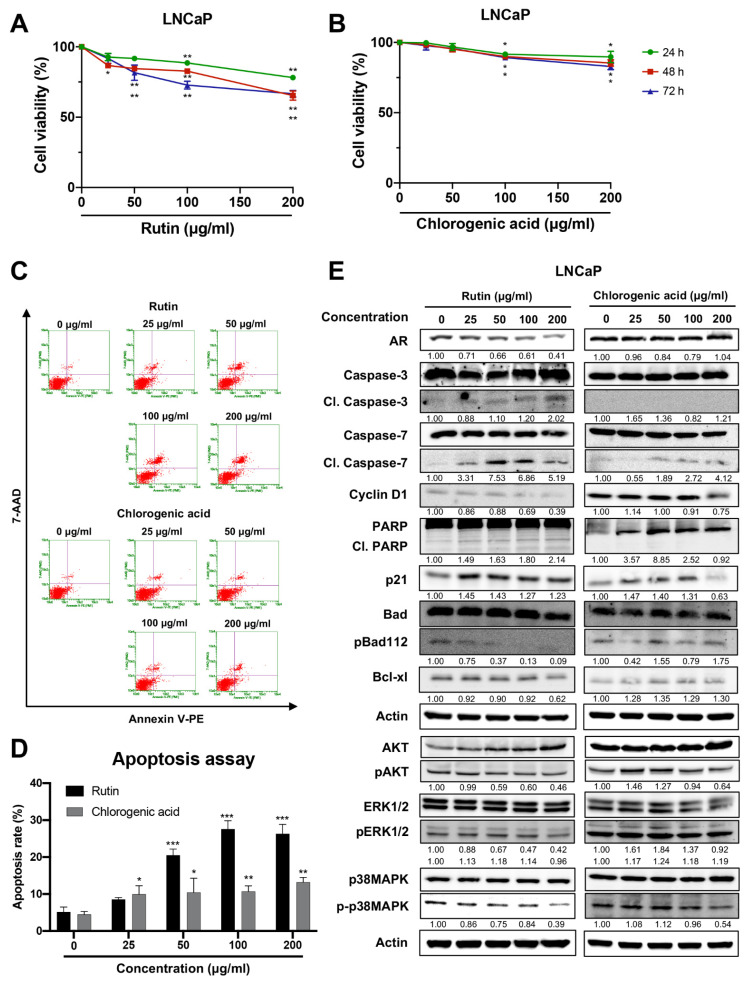
Effect of rutin and chlorogenic acid on apoptosis induction in LNCaP cells. The cytotoxic effect of rutin (**A**) and chlorogenic acid (**B**) on LNCaP cells was determined at 24, 48, and 72 h. Graphic dot plots of apoptotic cells (**C**) were generated and the percentage of total apoptotic cells (**D**) for LNCaP cells was quantitated in bar graphs. Immunoblotting analysis to show cell proliferation and apoptosis-related proteins in LNCaP cells in response to rutin and chlorogenic acid (**E**). Whole un-cropped images see Figure S5. Data represent mean ± SD. * *p* < 0.05, ** *p* < 0.01, and *** *p* < 0.001 vs. control.

To investigate the effect of the major compounds in HCT [17], LNCaP cells were treated with rutin or chlorogenic acid. Rutin diminished the viability of LNCaP cells in a dose-dependent manner (Figure 5A), and it increased cell apoptotic as indicated by flow cytometry (Figure 5C,D). Moreover, rutin also increased protein expression of cleaved caspases-3 and -7, cleaved PARP, and p21, while it decreased cyclin D1, p-Bad, and Bcl-xl protein expressions. Additionally, downstream signaling proteins such as AR, p-AKT, p-ERK1/2, and p-p38 MAPK were reduced by rutin (Figure 5E). In contrast, chlorogenic acid caused a slight but significant decrease in cell viability (Figure 5B) and weakly induced apoptosis, as determined by flow cytometry and immunoblotting in LNCaP cells (Figure 5C–E). These data suggest that rutin, the major compound in HCT, contributes to the stimulation of apoptosis in LNCaP cells.

### 3.5. HCT and EA Suppress EMT and Prostate Cancer Cell Migration through STAT3/Snail/Twist Pathways

**Figure 6:**
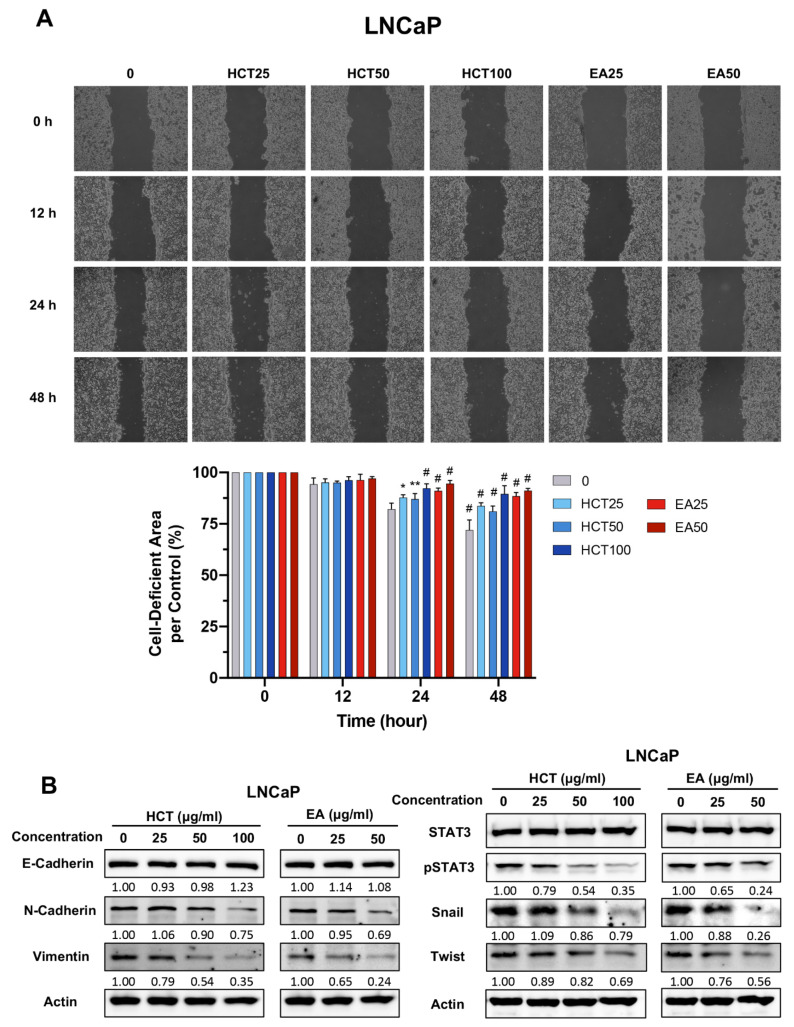
Effect of HCT and EA on cell migration and EMT phenotypes in LNCaP cells. Wound healing assay in LNCaP cells treated with HCT and EA for 12, 24, and 48 h, and the relative cell-deficient area to non-treated control group are represented in bar graphs (**A**). Immunoblotting of EMT-related proteins after HCT and EA treatment in LNCaP cells (**B**). Whole un-cropped images see Figure S6. Values represent mean ± SD from three independent experiments. * *p* < 0.05, ** *p* < 0.01, and ^#^ *p* < 0.001 vs. control. HCT, *Houttuynia cordata* Thunb.; EA, ethyl acetate fraction of *H. cordata*.

LNCaP cells are a prostate cancer cell line that can develop into more aggressive cancers by changing particular features, including migration [34]. We therefore investigated whether HCT and EA suppressed metastatic behavior by preventing cell migration in LNCaP cells. The migration potential of LNCaP cells was examined in the serum-free condition to avoid the influence of cell proliferation. HCT (100 μg/mL) and EA (50 μg/mL) slightly decreased cell viability to approximately 80% as compared to non-treated control in LNCaP cells at 48 h (Supplementary Figure S1). The wound healing assay indicated that the cell-deficient areas in HCT (100 μg/mL) and EA (50 μg/mL) treatments were maintained in 89.6 ± 4.0% and 91.1 ± 1.1%, respectively, as compared with the non-treated control (71.9 ± 5.0%) at 48 h (Figure 6A). These data suggest that HCT and EA have at least a weak inhibitory effect on cell migration in LNCaP cells. The activation of the STAT3/Twist pathway in prostate cancer cells promotes epithelial–mesenchymal transition (EMT), allowing the cells to migrate [35,36,37]. We were interested in the effect of HCT and EA on STAT3-regulated EMT in LNCaP cells. Remarkably, the expression of EMT-related proteins such as N-cadherin and vimentin was decreased by the treatments. Similarly, downstream proteins of the EMT signaling pathway, including p-STAT3, Snail, and Twist, were reduced in both HCT-treated and EA-treated LNCaP cells (Figure 6B). These results indicate that HCT and EA inhibit cell migration and EMT phenotypes via STAT3/Snail/Twist pathways in prostate cancer cells.

### 3.6. Suppression of Prostate Carcinogenesis by HCT in the TRAP Model

**Figure 7:**
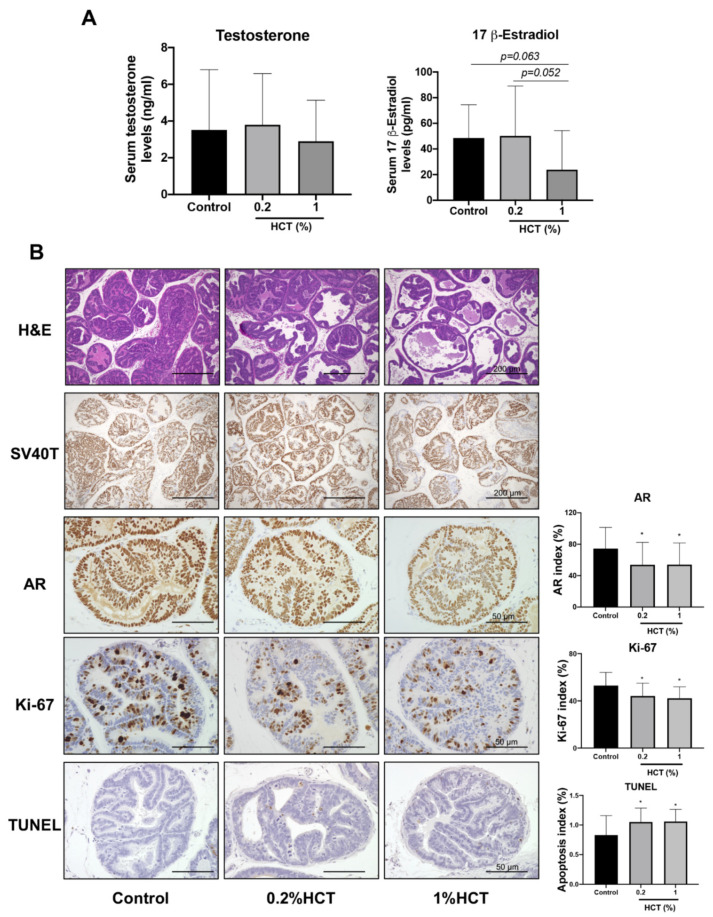
Effect of HCT on prostate carcinogenesis in the TRAP model. Testosterone and 17 β-estradiol level determined by competitive enzyme-linked immunosorbent assay (**A**) and representative images of H&E staining, immunohistochemistry of androgen receptor (AR), Ki-67 and terminal deoxynucleotidyl transferase dUTP nick end labeling (TUNEL) assay in high grade prostatic intraepithelial neoplasia in prostate lateral lobe (**B**). * *p* < 0.05 vs. control group. HCT, *Houttuynia cordata* Thunb.; TRAP, Transgenic Rat for Adenocarcinoma of Prostate.

The inhibitory effect of HCT and EA on prostate cancer in vitro suggests that they may have a chemopreventive effect on prostate carcinogenesis in vivo. The amount of EA obtained from HCT was limited; 1 kg of *H. cordata* yielded about 130 g of HCT crude or 16.6 g of EA. Therefore, we next investigated the effect of HCT on the rat androgen-sensitive prostate cancer model, TRAP. HCT extract did not appear to produce any toxicity or adverse effect in the body, nor did it affect organ weight and serum levels of testosterone and estrogen at the end of the experiment; average HCT consumption in the 0.2% and 1% HCT mixed diet group was about 115.9 ± 2.2 and 589.2 ± 12.7 mg/kg/day, respectively (Table 2 and Figure 7A). In both the lateral and ventral prostate, the percentage of LG-PIN in the high-dose groups was significantly increased relative to the control group (Table 3). The percentage of adenocarcinoma in the lateral prostate was markedly decreased in the HCT-fed group, while that in the ventral prostate was significantly decreased only in the 0.2% HCT treatment group. The incidence of adenocarcinoma in lateral prostate was significantly decreased in both the 0.2% and 1% HCT-fed groups (Table 3). Moreover, the significantly decreased percentages of AR and Ki-67 positive cells were subjected to immunohistochemical examination in the lateral lobe of prostates in HCT-fed groups (Figure 7B) and there were also significantly increased apoptotic cells in the lateral prostate of the HCT-fed groups. These changes were not observed in the ventral prostate of the HCT-fed groups (Supplementary Figure S2). Thus, using the TRAP model we found that HCT extract has an inhibitory effect on prostate carcinogenesis through not only inhibition of cell proliferation but also an increase of apoptosis without any adverse effects.

### 3.7. HCT Triggers Apoptosis and Suppresses Cell Proliferation via AKT/ERK/p38 MAPK Pathways in the TRAP Model

**Figure 8:**
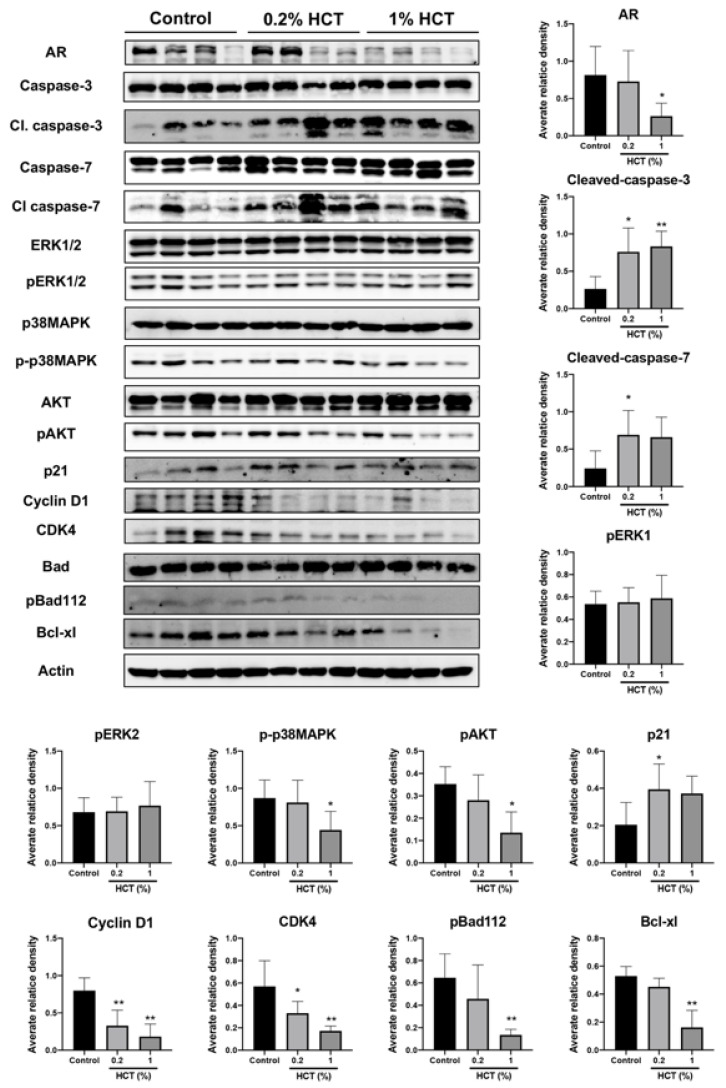
Effect of HCT on protein expression in the TRAP model. Immunoblotting and average relative band density for cell proliferation and apoptosis-related proteins in prostate lateral lobe. Whole un-cropped images see Figure S7. Bar graphs represent the mean ± SD. * *p* < 0.05 and ** *p* < 0.01 vs. control. HCT, *Houttuynia cordata* Thunb.; TRAP, Transgenic Rat for Adenocarcinoma of Prostate.

HCT decreased AR protein expression and upstream signaling proteins in our in vitro study, and we determined whether HCT also has an inhibitory effect in vivo using the lateral lobe of the prostate. HCT decreased the expression of proteins involved in cell growth and cell cycle pathways, including AR, cyclin D1, CDK4, p-p38 MAPK, and p-AKT (Figure 8). In addition, apoptosis-related proteins such as caspases-3 and -7 were significantly increased by HCT treatment. These results indicate that apoptotic and anti-proliferative effects of HCT and EA treatment observed in LNCaP cells similarly occurred in the TRAP model via inhibition of the AKT and p38 MAPK signaling pathways.

### 3.8. HCT Inhibit Tumor Growth and Their Signaling in the PCai1 Xenograft Mice Model

**Figure 9:**
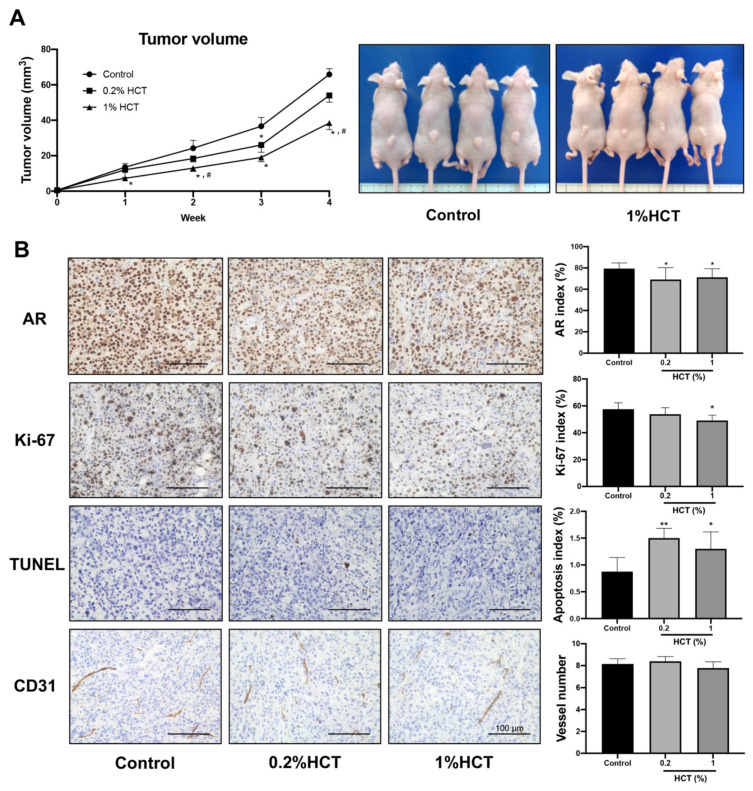
Effect of HCT on castration-resistant prostate cancer in the PCai1 xenograft model. Tumor volume of PCai1 xenograft mice that received control diet or HCT mixed diet (**A**). Representative images of immunohistochemistry for androgen receptor (AR), Ki-67, terminal deoxynucleotidyl transferase dUTP nick end labeling (TUNEL) assay, and the blood vessel marker, CD31, in PCai1 tumor (**B**). Data in bar graphs represent mean ± SD. * *p* < 0.05, ** *p* < 0.01 vs. control group. # *p* < 0.05 vs. 0.2% HCT group. HCT, *Houttuynia cordata* Thunb.

**Figure 10:**
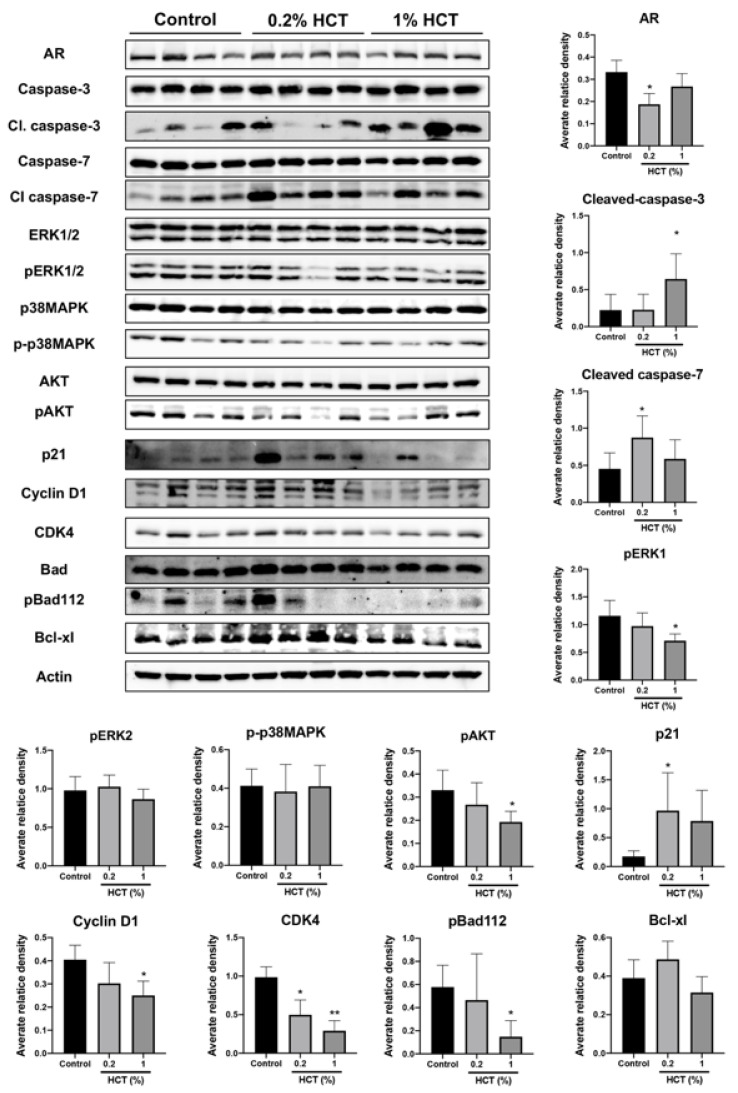
Effect of HCT on protein expression in the PCai1 xenograft mice model. Immunoblotting and average relative band density for cell proliferation and apoptosis-related proteins in the PCai1 xenograft mice model. Whole un-cropped images see Figure S8. Bar graphs represent means ± SD. * *p* < 0.05 and ** *p* < 0.01 vs. control. HCT, *Houttuynia cordata* Thunb.

Apoptosis induction triggered by HCT and the molecular mechanism in prostate cancer cells in vitro were similar between androgen-sensitive and CRPC cells. We further examined the effect of HCT on CRPC tumors using the PCai1 xenograft model. Mice in 0.2% and 1% HCT groups received 25.2 ± 0.3 and 127.0 ± 1.9 mg/kg/day of HCT, respectively. HCT showed no signs of toxicity, as no changes in body and organ weights were observed in either group (Table 4). As shown in Figure 9A, the tumor volume in mice fed 1% HCT mixed diet was significantly lower than that in the control diet group. Moreover, the results indicate that both AR and Ki-67 indices were significantly decreased in PCai1 tumors of the HCT-fed groups as compared to those in the control group. Importantly, apoptotic cells were significantly increased in PCai1 tumors following HCT treatment. However, there was no significant difference in vessel number between the control and HCT-fed groups (Figure 9B). Hence, HCT extract inhibited tumor growth via induction of apoptosis in the PCai1 xenograft model. Comparing with the protein expression changes induced by HCT treatment in the TRAP model, these results indicate that the AR, cyclin D1, CDK4, p-AKT, and p-ERK1 were downregulated, while cleaved caspases-3 and -7, and p21, were upregulated (Figure 10). Therefore, the results suggest that HCT has a potent inhibitory effect on cell proliferation and apoptosis induction in the PCai1 xenograft mouse model.

## 4. Discussion

Medicinal plants, such as HCT are used in many health maintenance products and basic dietary supplements. Studies on the effect of regular consumption of medical plants have yielded interesting results; for example, consuming three tablespoons of flaxseed per day was shown to improved cancer cell clearance and reduce the rates of cell proliferation in prostate cancer [38]. We previously demonstrated that the inhibitory effect of HCT on cell viability in breast cancer cell lines was much stronger than that in normal cells, human mammary epithelial cell line (MCF10A), and human peripheral blood mononuclear cells (PBMCs) [17]. In addition, in our present study, HCT extract did not show any signs of adverse effects in in vivo studies; there was no substantial difference in body, organ weights, and histology between the control and HCT-treated groups (Table 2 and Table 4). These studies suggest that HCT is more sensitive to cancer cells than normal cells. These results suggest that certain plant-based foods containing phytonutrients may be useful as cancer chemopreventive agents. Due to the phytochemical components in HCT [16,39], our previous study using HPLC demonstrated that rutin is the major compound in HCT [17]. Rutin and chlorogenic acid were reported to inhibit cell proliferation, and migration on human prostate cancer cells, even though these compounds are derived from different kinds of herbs [40,41,42]. Rutin also complements other active ingredients such as chemotherapeutic drugs and herbal extracts [43], and additionally, at low concentrations, is combined with the chemotherapeutic drugs to evaluate the synergistic efficiency of the combination against cancer cells [44,45]. Chlorogenic acid has also been reported to have a potent anti-cancer effect against several types of cancer cells, including human lung cancer [46]. In the present study, HCT treatment inhibited activation of AKT signaling, resulting in inducing caspase-dependent apoptosis in prostate cancer cells. Similar to HCT, rutin suppressed cell viability by inactivation of AKT and induction of caspase-dependent apoptosis in prostate cancer cells. On the other hand, the growth inhibitory effect of chlorogenic acid was limited to weak in LNCaP cells. These results suggest that rutin is one of the active components of HCT as summarized in Graphic Abstract. Compared with the cytotoxicity effect and flow cytometric analysis of apoptosis in this study, the effect of rutin tended to be weaker than that of the crude extract, HCT, in LNCaP cell lines. Likewise, previous studies indicated that the additive and synergistic effects of phytochemicals in fruit and vegetables are involved in their anticancer properties [47,48,49]. We found other phenolic acid and flavonoids as minor components in EA fraction. The interaction of all components, including minor components, is worth investigating in further studies; however, these effects do not occur when the constituent compounds are ingested in purified form; Hence, we focus on consumption of whole fruits and vegetables rather than their isolated constituents.

We initially investigated the effect of HCT and EA on apoptosis induction using androgen-sensitive prostate cancer and CRPC cell lines. PCai1 cells, which were established initially from castration-resistant prostate tumors, can be grown in androgen-free medium conditions, and the normal AR function is maintained [24,50]. Previous studies have shown that HCT extract induces apoptosis in various cancer cell types, including human lung cancer A549 cells, by activating caspase-8 and caspase-3 [51]. However, in-depth studies of the plant on the mechanism of action in prostate cancer have not yet been performed. Our results revealed that HCT and EA activated cleaved caspase-3 and -7 protein expression in a dose-dependent manner, and they also down-regulated AKT, ERK, and MAPK-signaling pathway function cooperatively to promote prostate tumorigenicity and androgen independence [26,52,53]. The AKT pathway can be activated by various growth factors and plays a crucial role in promoting cell growth and blocking apoptosis in various cancer models, including prostate cancer, and it has already been reported that activated AKT phosphorylates several apoptosis-regulating proteins including BAD, a member of the pro-apoptotic BH3-only Bcl-2 family in prostate cancer cells [54,55]. Activation of AKT or ERK plays an important role in enhancing cell proliferation and inhibiting apoptosis [56]. Therefore, some agents that suppress these signaling molecules are useful for chemoprevention. For example, resveratrol regulates the AKT and ERK pathways through androgen receptor-independent mechanisms in prostate cancer cells and also inhibits AR pathways in androgen receptor-dependent prostate cancer cells [57]. Our findings demonstrate that the decreased expression of p-AKT, p-ERK1/2, and p-p38 MAPK by HCT or EA induced apoptosis in both LNCaP and PCai1 cells. These effects also led to the reduction of cyclin D1 and CKD4 and an increase in p21 protein expression levels. Our results suggest the involvement of phosphorylation in protein kinase pathways, including, AKT, ERK and p38 MAPK in regulating the induction of apoptosis by HCT and EA treatment in these two different types of prostate cancer cells.

During prostate carcinogenesis, EMT is related to cancer progression, migration and metastasis, and mesenchymal markers and transcription factors, such as vimentin, N-cadherin, Snai1, and Twist1/2, are highly upregulated [58]. Epithelial cell markers such as E-cadherin are decreased, leading to loss of cell–cell adhesion, whereas mesenchymal markers such as vimentin and N-cadherin are increased, thus permitting the cells to migrate to secondary sites or organs. These can be considered molecular targets for inhibiting EMT in prostate cancers. In a previous study, suppression of EMT by arenobufagin decreased cell migration and invasion in PC3 cells [59]. We found HCT and EA inhibited the expression of mesenchymal biomarkers such as N-cadherin and vimentin in LNCaP cells. Nevertheless, no alteration of E-cadherin was observed. In addition, both extracts reduced the protein expression of phosphorylated STAT3, Snail, and Twist, which are related to the EMT signaling pathway in prostate cancers [60]. These results indicate HCT and EA inhibited EMT phenotype. LNCaP cells expressed both epithelial and mesenchymal makers and have heterogenous population in normal condition [61]. According to this knowledge, inhibition of vimentin and N-cadherin HCT and EA indicates a decrease of cells with mesenchymal phenotype in LNCaP. It may induce an increase of cells with epithelial phenotype. Therefore, we consider that HCT and EA induced the reversal of EMT phenotype, and did not selectively eliminate post-EMT cells in LNCaP. However, further studies using flowcytometry are needed. Due to the spheroid formation of PCai1 cells, wound healing assay was not suitable for studying cell migration, and we therefore conducted such experiments in LNCaP cells only. Interestingly, the AKT and ERK signaling pathway affects EMT, causing it to activate cancer cell migration [62,63]. In the present study, HCT and EA decreased phosphorylation of AKT and also inhibited EMT and cell migration. These results indicate that HCT and EA may repress EMT-associated cell migration via inactivation of AKT or ERK, and at least are involved in decreased motility of prostate cancer cells.

The HCT extract was mixed with food to mimic the daily food consumption. Likewise, many in vivo studies have reported the use of compound-mixed diets [64,65]. These studies clearly showed that HCT increased LG-PIN and decreased adenocarcinoma in the lateral lobe of the prostate gland in the TRAP model. According to results from both TRAP and CRPC xenograft models, HCT induced caspase-dependent apoptosis in prostate cancer and also inhibited cell proliferation, while decreasing expression of cell proliferation-related proteins. High labeling index of Ki-67, a biomarker of cell proliferation, is associated with poor prognosis of prostate cancer [66,67]. In addition, not only an increase of Ki-67 but also a decrease of apoptosis occurred in prostate cancer tissues [68]. Analysis of the AR labeling index has demonstrated a critical association between Ki-67 indices and AR expression in more rapidly proliferating cells [69]. In this immunohistochemical analysis, the AR and Ki-67 indexes decreased, whereas increased apoptosis occurred with 1% HCT in both experimental animal models. This indicates that a potent anti-proliferative effect may provide chemopreventive properties by inducing apoptosis.

Our study is non-clinical research, which investigated the inhibitory effect of HCT on prostate carcinogenesis and CRPC using in vitro and in vivo models. A few clinical studies reported safety of HCT and beneficial effects on other diseases [70,71,72]. Even though there are a limited number of clinical studies related to HCT, we believe that evidence from this study may lead to future clinical studies to investigate chemopreventive effects of HCT on prostate carcinogenesis.

## 5. Conclusions

In this study, we analyzed the effect of HCT on several stages of prostate cancer. HCT suppressed both prostate carcinogenesis in the TRAP model and CRPC tumor growth in the xenograft model. Hence, we assumed that the inhibitory mechanism of HCT on both carcinogenesis and castration-resistant phenotypes of prostate cancer may be explained by the same mechanism inducing caspase-dependent apoptosis and inhibiting cell growth-related proteins via inactivation of AKT/ERK/MAPK. From all these studies, HCT is expected to be effective against prostate carcinogenesis and CRPC.

## Figures and Tables

**Table 1:** Chemical components of HCT (*Houttuynia cordata* Thunb.) and its fractions.

Compounds	*H. cordata*	Hexane Fraction	Ethyl Acetate Fraction	Residue
Gallic acid	0.44 ± 0.01	ND	0.64 ± 0.05	ND
Catechin	1.64 ± 0.43	ND	0.50 ± 0.15	ND
Chlorogenic acid	25.50 ± 3.41	ND	15.15 ± 1.37	3.68 ± 0.15
Vanilic acid	0.84 ± 0.14	ND	0.10 ± 0.04	0.15 ± 0.02
Ferulic acid	0.62 ± 0.02	ND	1.51 ± 0.18	ND
*p*-Courmaric acid	0.12 ± 0.02	ND	0.11 ± 0.24	ND
Rutin	44.00 ± 5.61	ND	81.3 ± 5.21	2.40 ± 0.27
Rosmarinic acid	1.49 ± 0.04	ND	1.97 ± 0.74	ND
Quercetin	0.20 ± 0.05	ND	ND	ND
Apigenin	ND	ND	ND	ND

The data are expressed as mean ± SD of three independent experiments; ND = not detected.

**Table 2:** Body, organ weights, and average HCT intake of TRAP rat fed with HCT.

Trait	Group		
	Control	0.2%HCT	1%HCT
No. of rats	13	12	12
Initial body weight (Day 1) (g)	244.9 ± 6.6	248.1 ± 7.3	250.2 ± 8.6
Final body weight (Day 70) (g)	645.0 ± 21.5	664.7 ± 15.3	656.7 ± 28.9
Average food intake (g/rat/day)	25.5 ± 1.2	25.5 ± 1.1	26.0 ± 1.3
Average HCT intake (mg/kg/day)	0.0	115.9 ± 2.2	589.2 ± 12.7
Organ weight (g)			
Liver	23.38 ± 2.61	21.31 ± 1.80	22.82 ± 2.90
Kidneys	3.06 ± 0.12	3.07 ± 0.11	3.12 ± 0.09
Prostate glands	0.25 ± 0.04	0.30 ± 0.05	0.29 ± 0.05

Data are shown as the mean ± SD. TRAP, Transgenic Rat for Adenocarcinoma of Prostate; HCT, *Houttuynia cordata* Thunb.

**Table 3:** Incidence of carcinoma and the percentage of prostatic neoplastic lesions in TRAP rat treated with HCT.

Treatments	No. of Rats	Incidence of Adenocarcinoma (%)	Percentage of Lesion in Prostate		
			LG-PIN	HG-PIN	Adenocarcinoma
Lateral Lobe					
Control	13	13 (100%)	4.72 ± 2.95	89.18 ± 3.04	6.06 ± 2.69
0.2%HCT	12	8 (67%) *	7.60 ± 4.48	90.55 ± 5.41	2.22 ± 1.96 ***
1%HCT	12	6 (50%) **	10.31 ± 4.76 **	88.38 ± 4.18	1.32 ± 1.65 ***
Ventral Lobe					
Control	13	13 (100%)	4.61 ± 1.53	89.70 ± 2.13	5.66 ± 2.42
0.2%HCT	12	11 (92%)	6.76 ± 2.89 *	90.91 ± 2.64	2.32 ± 1.51 ***
1%HCT	12	11 (92%)	8.82 ± 3.80 **	87.87 ± 3.55	3.33 ± 3.38

TRAP, Transgenic Rat for Adenocarcinoma of Prostate; HCT, *Houttuynia cordata* Thunb.; LG, low grade; HG-PIN, high grade prostatic intraepithelial neoplasia. The data are expressed as mean ± SD. * *p* < 0.05; ** *p* < 0.01; *** *p* < 0.001 compared to control group using one-way ANOVA (post hoc: LSD).

**Table 4:** Body weight, organ weight, and average HCT consumption in mice treated with HCT.

Trait	Group		
	Control	0.2% HCT	1% HCT
Initial body weight (Day 1) (g)	25.9 ± 0.7	25.8 ± 0.2	25.9 ± 0.6
Final body weight (Day 28) (g)	27.7 ± 0.1	27.9 ± 0.6	27.8 ± 0.5
Average food consumption (g/mouse/day)	3.4 ± 0.2	3.5 ± 0.1	3.5 ± 0.2
Average HCT consumption (mg/kg/day)	0.0	25.2 ± 0.3	127.0 ± 1.9
Organ weight (g)			
Liver	1.51 ± 0.07	1.49 ± 0.03	1.51 ± 0.04
Kidneys	0.48 ± 0.01	0.49 ± 0.01	0.49 ± 0.01

HCT, *Houttuynia cordata* Thunb.

## Data Availability

The data presented in this study are available on request from the corresponding author.

## Supplementary Materials

The following are available online at https://www.mdpi.com/article/10.3390/cancers13143403/s1, Figure S1:, Cytotoxic effects of HCT and EA in LNCaP cells in serum-free condition, Figure S2: Representative images of H&E staining, immunohistochemistry of androgen receptor (AR), Ki-67 and terminal deox-ynucleotidyl transferase dUTP nick end labeling (TUNEL) assay in high grade prostatic intraepithelial neoplasia in prostates ventral lobe, Figures S3–S8: Whole un-cropped images of the original western blots showing all bands with molecular weight markers.

## Abbreviations

The following abbreviations are used in this manuscript:

ADT	Androgen deprivation therapy
AKT	Protein kinase B
AR	Androgen receptor
BPH	Benign prostatic hyperplasia
CRPC	Castration-resistant prostate cancer
DMSO	Dimethyl sulfoxide
EA	Ethyl acetate fraction of *H. cordata*
EMT	Epithelial-mesenchymal transition
ERK	Extracellular signal-regulated kinase
FBS	Fetal bovine serum
H	Hexane fraction of *H. cordata*
HCT	*Houttuynia cordata* Thunb.
HG-PIN	High-grade prostatic intraepithelial neoplasia
H&E	Hematoxylin and eosin
IHC	Immunohistochemical analysis
LG-PIN	Low-grade prostatic intraepithelial neoplasia
p38 MAPK	p38 mitogen-activated protein kinase
PARP	Poly (ADP-ribose) polymerase
R	Residue fraction of *H. cordata*
TRAP	Transgenic rat for adenocarcinoma of prostate
TUNEL	Terminal deoxynucleotidyl transferase dUTP nick end labelling
