# Supplementary material for: Suppressive Effect and Molecular Mechanism of Houttuynia cordata Thunb. Extract against Prostate Carcinogenesis and Castration-Resistant Prostate Cancer
Source: Cancers (Basel). 2021 Jul 7;13(14):3403. doi: 10.3390/cancers13143403 (PMC8306559; doi:10.3390/cancers13143403)
Supplement: Supplementary file 1 [file cancers-13-03403-s001.zip › Supplementary Materials_cancsers-1249782_revise.docx.pdf]

cancers

Supplementary Materials

Suppressive effect and molecular mechanism of *Houttuynia cordata* Thunb. extract against prostate carcinogenesis and castration-resistant prostate cancer

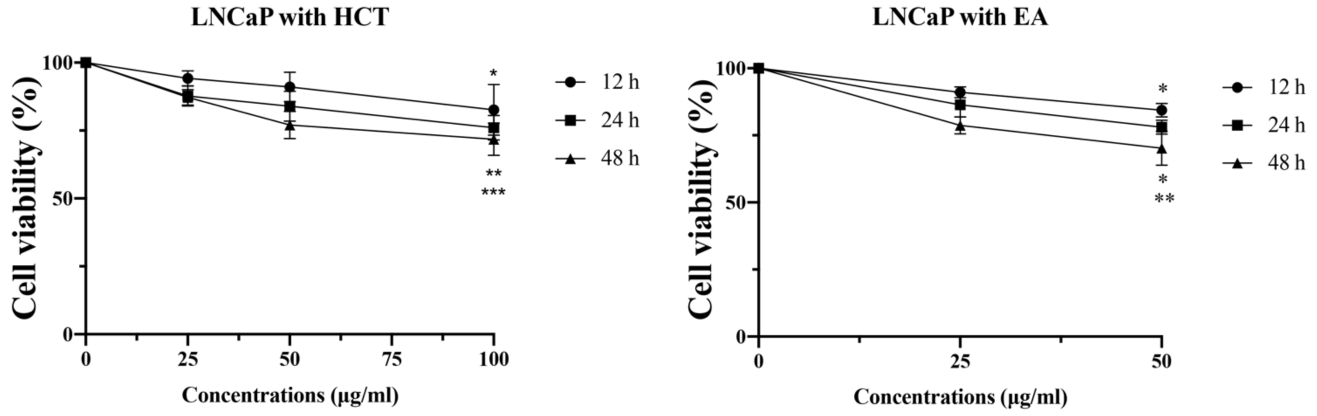

Supplementary Figure S1. Cytotoxic effects of HCT and EA in LNCaP cells in serum-free condition. LNCaP were treated with HCT (25-100 µg/mL) or EA (25-50 µg/mL) in incomplete media at 12, 24 and 48 h. The data represent 3 independent experiments. $*p < 0.05$; $**p < 0.01$; $***p < 0.001$ vs. control (one-way ANOVA with Tukey's post hoc test)

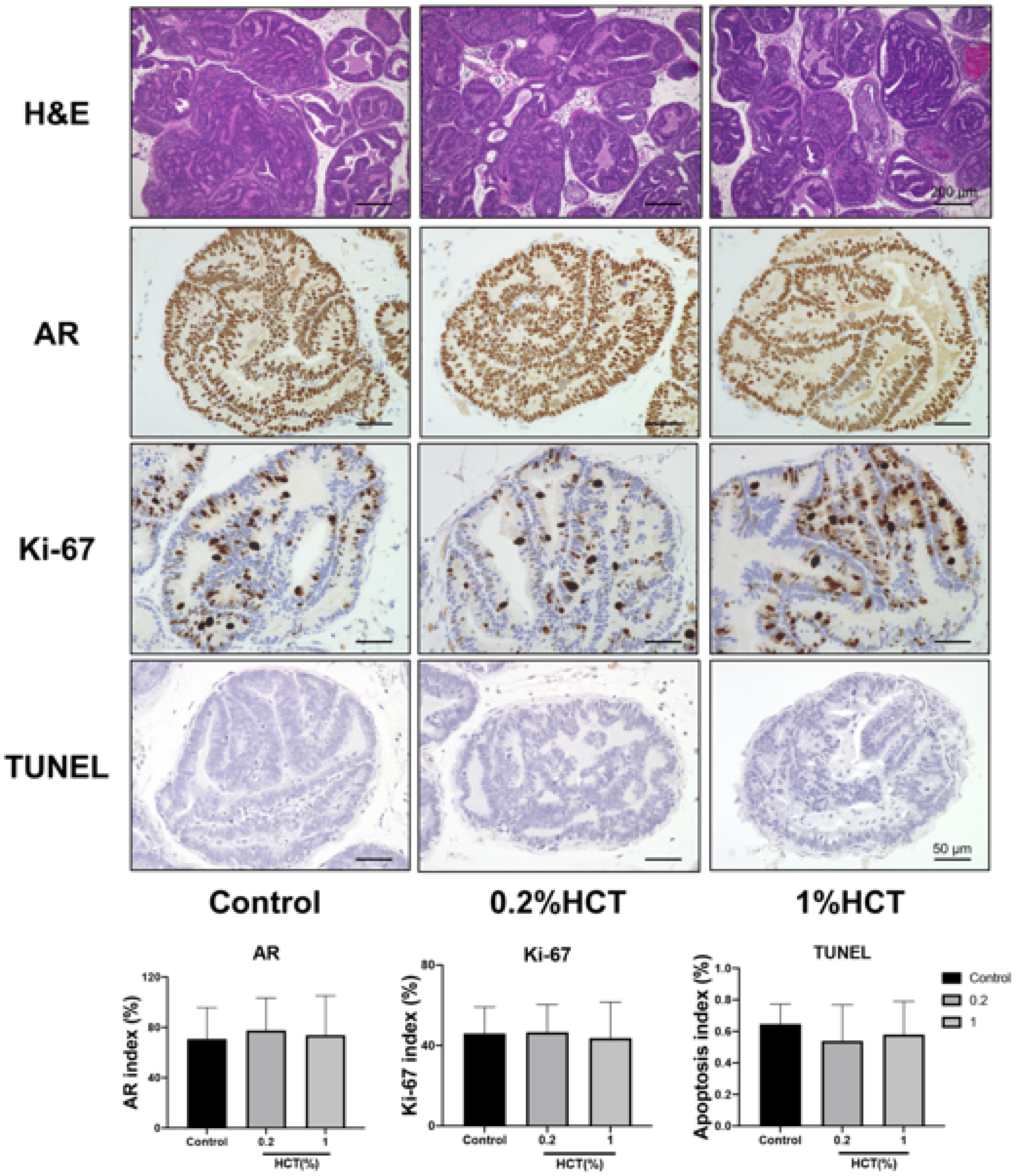

Supplementary Figure S2. Representative images of H&E staining, immunohistochemistry of androgen receptor (AR), Ki-67 and terminal deox-ynucleotidyl transferase dUTP nick end labeling (TUNEL) assay in high grade prostatic intraepithelial neoplasia in prostates ventral lobe.

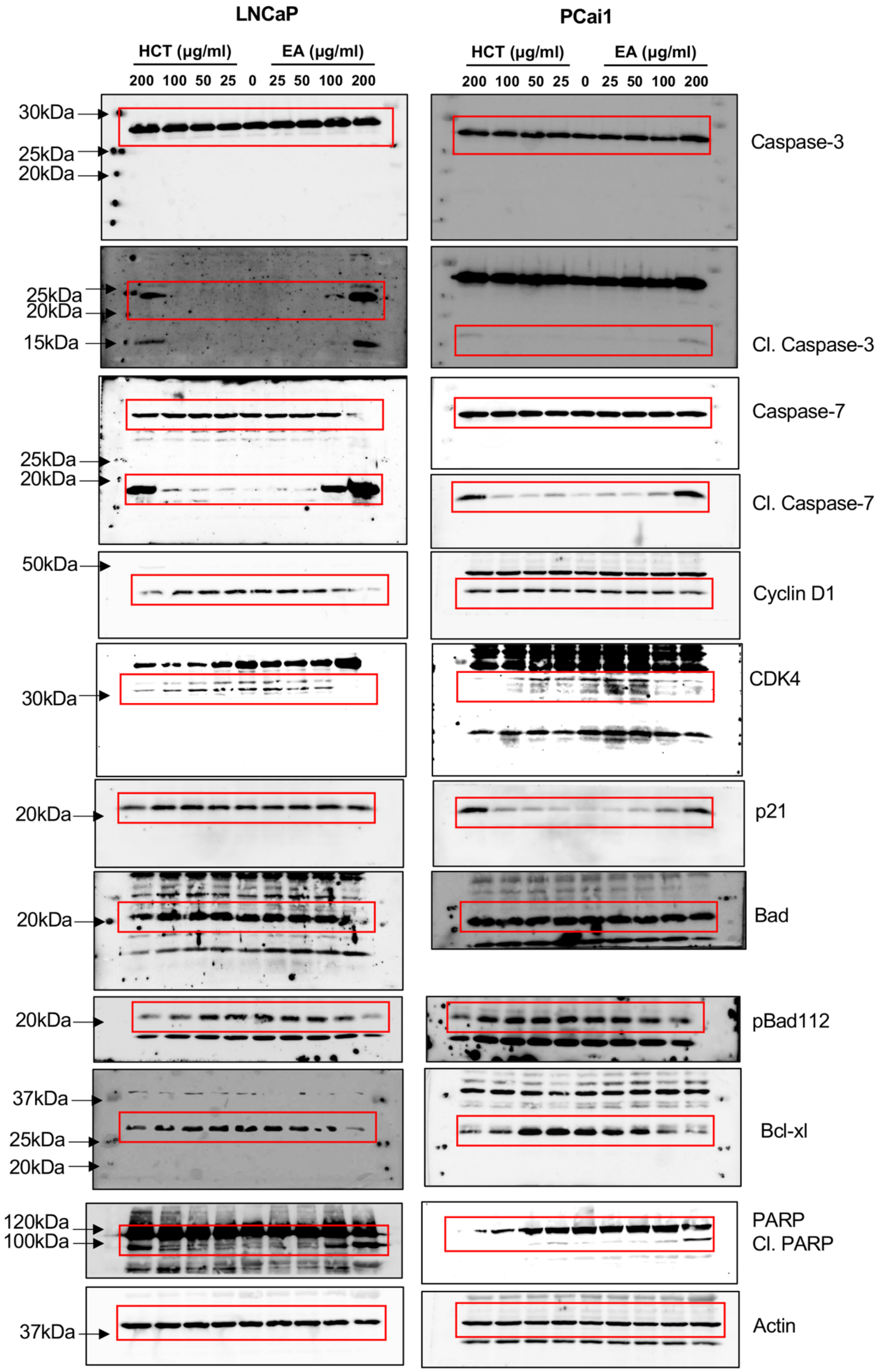

Supplementary Figure S3. Whole un-cropped images of the original western blots showing all bands with molecular weight markers. These gels correspond to those shown in Figure 3D

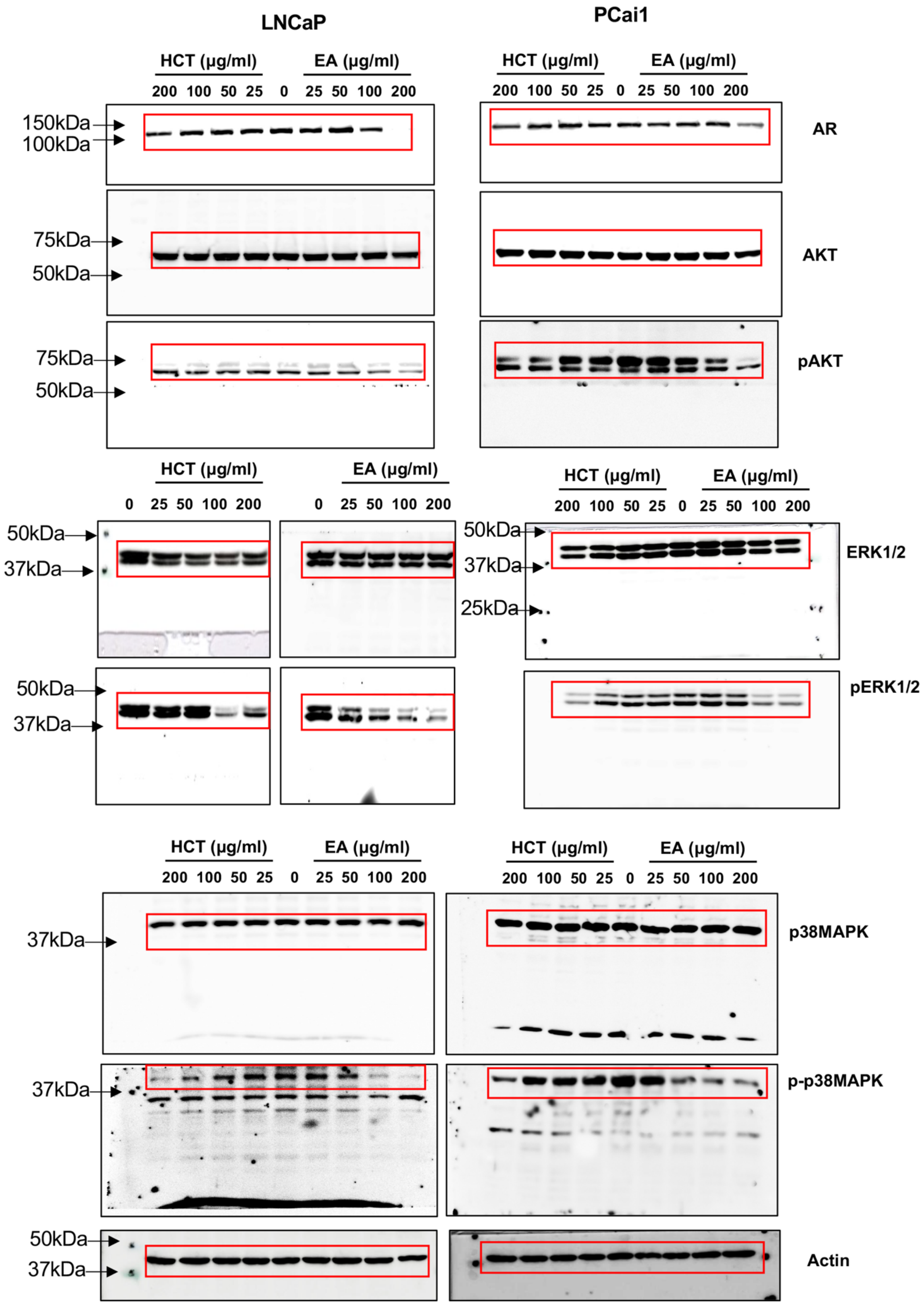

Supplementary Figure S4. Whole un-cropped images of the original western blots showing all bands with molecular weight markers. These gels correspond to those shown in Figure 4

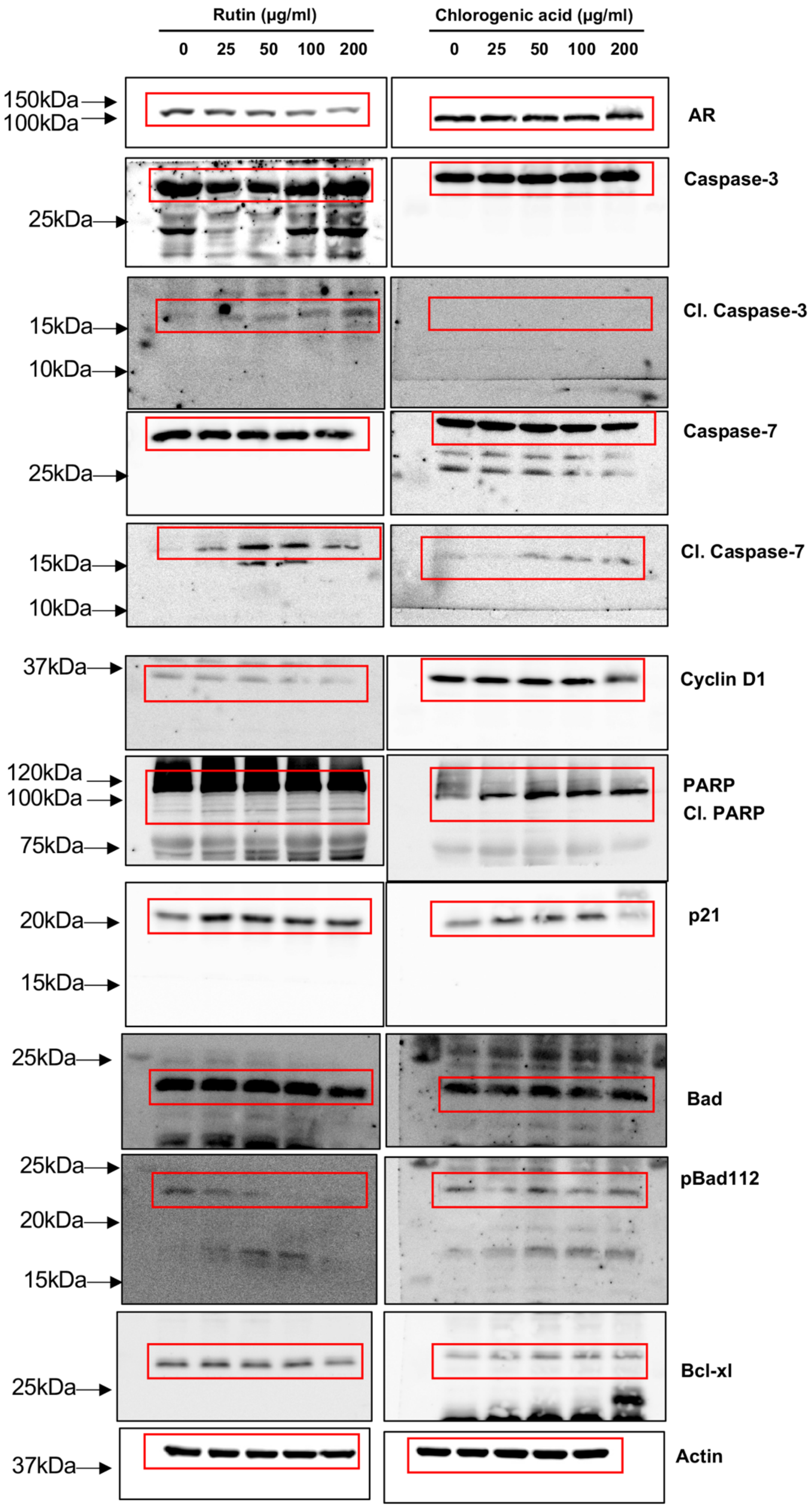

Supplementary Figure S5. *Cont.*

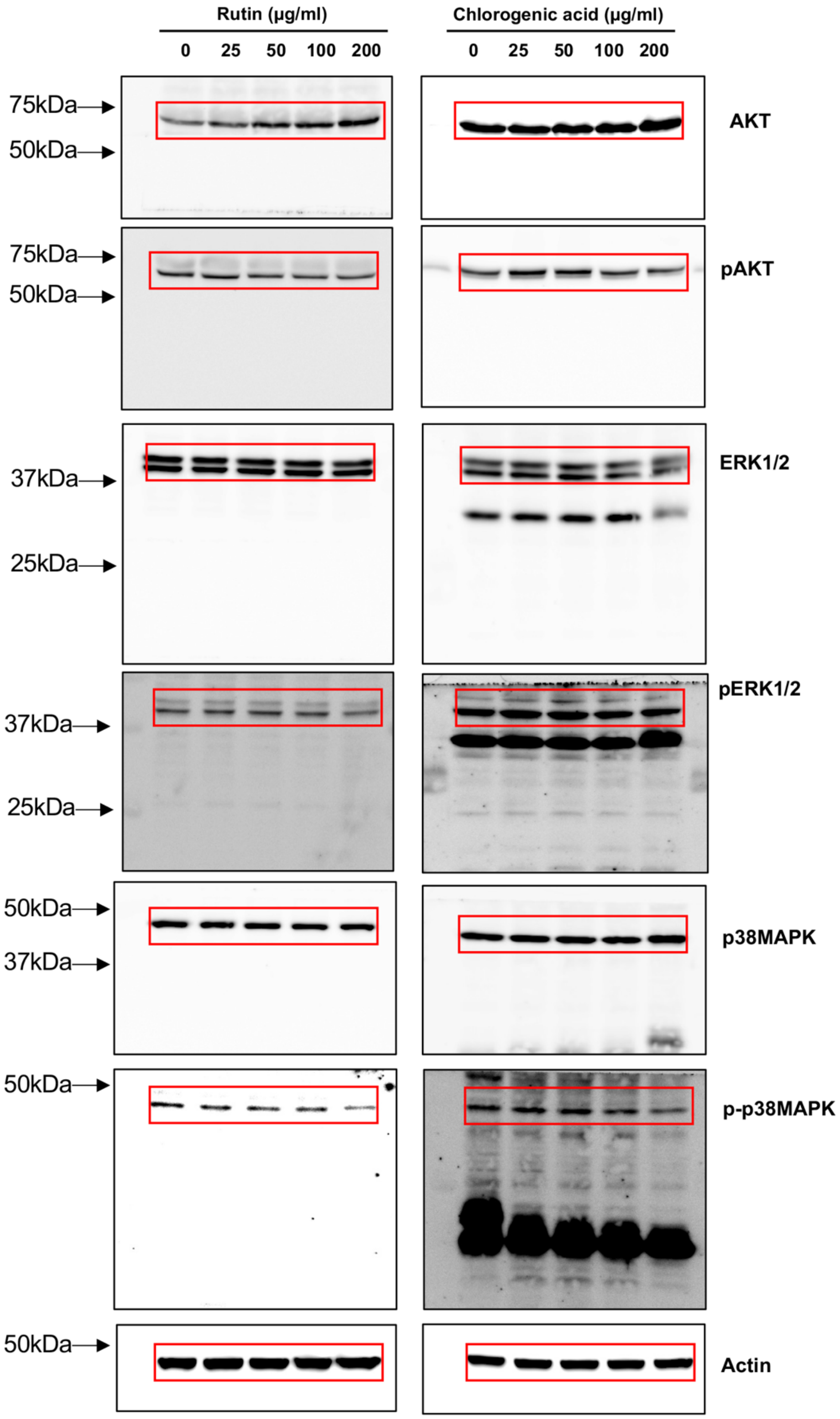

Supplementary Figure S5. Whole un-cropped images of the original western blots showing all bands with molecular weight markers. These gels correspond to those shown in Figure 5E

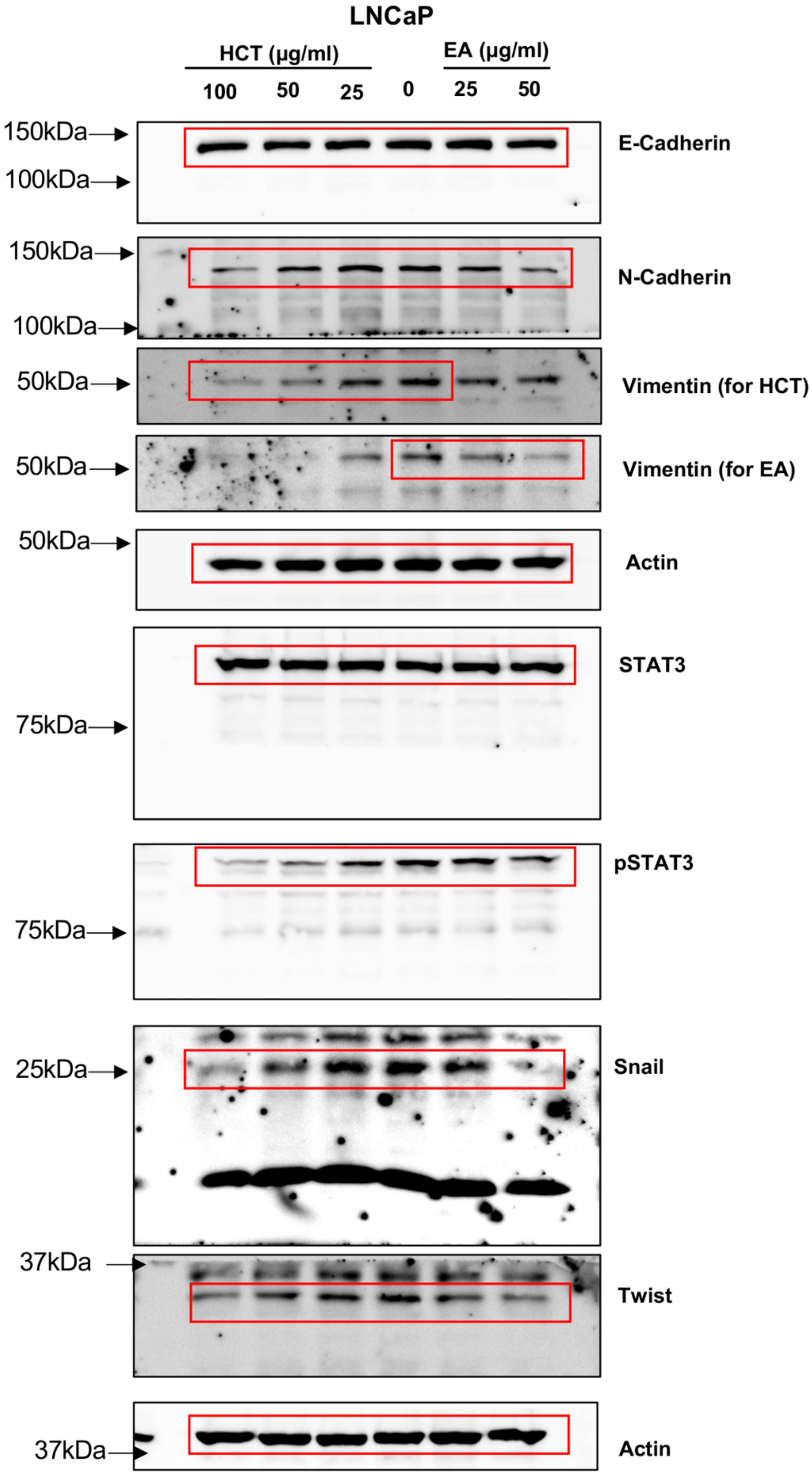

Supplementary Figure S6. Whole un-cropped images of the original western blots showing all bands with molecular weight markers. These gels correspond to those shown in Figure 6B

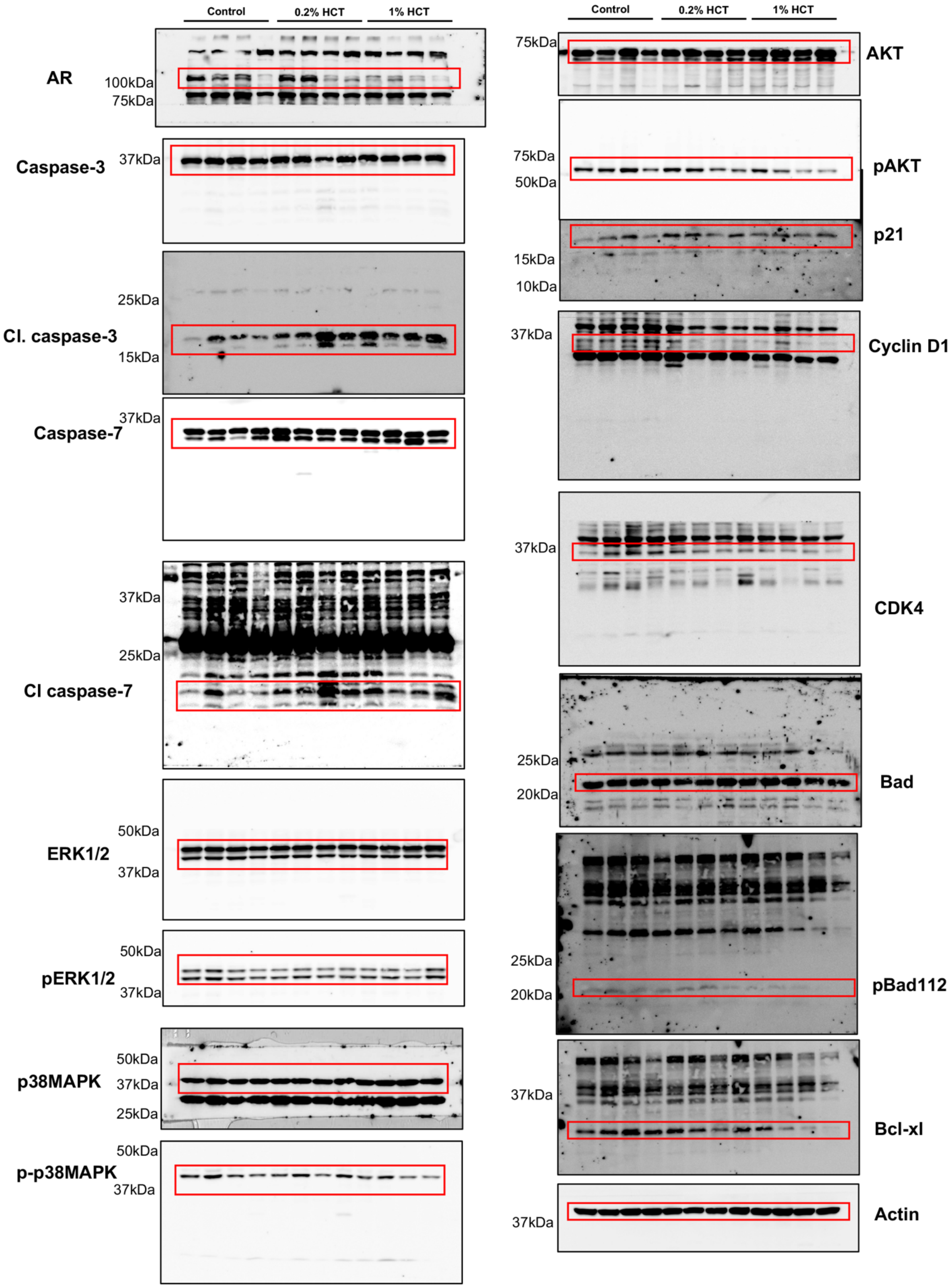

Supplementary Figure S7. Whole un-cropped images of the original western blots showing all bands with molecular weight markers. These gels correspond to those shown in Figure 8

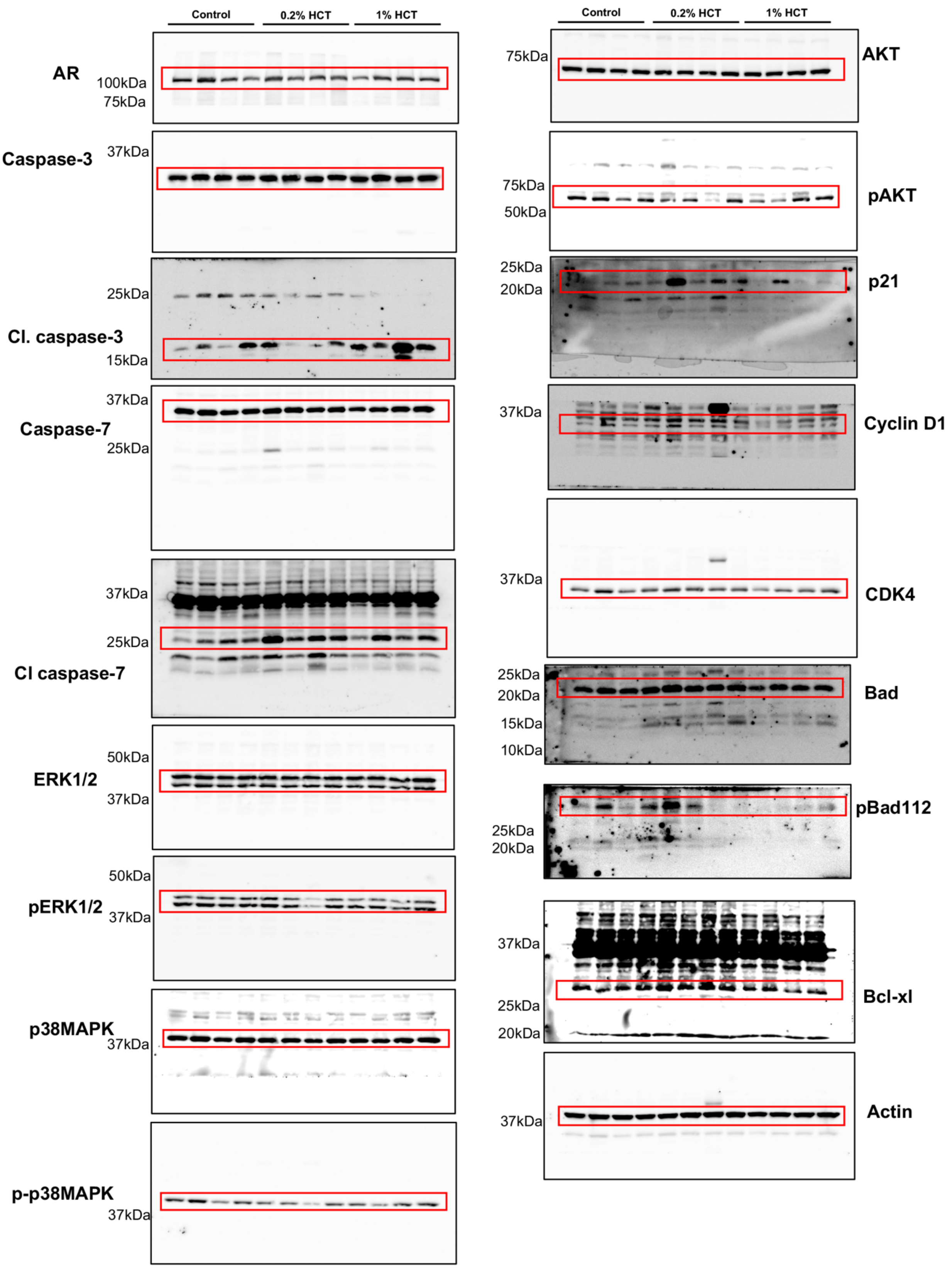

Supplementary Figure S8. Whole un-cropped images of the original western blots showing all bands with molecular weight markers. These gels correspond to those shown in Figure 10
